# Reconsidering the Role of Blood Cultures in Community‐Acquired Pneumonia: A Case of Co‐Infection With *Streptococcus pneumoniae* and *Klebsiella variicola*

**DOI:** 10.1002/rcr2.70457

**Published:** 2026-01-05

**Authors:** Naoki Fujimoto, Issei Oi, Kohei Fujita, Saiki Yoshimura, Takanori Ito, Takuma Imakita, Osamu Kanai, Kiminobu Tanizawa

**Affiliations:** ^1^ Division of Respiratory Medicine Center for Respiratory Diseases, National Hospital Organization Kyoto Medical Center Kyoto Kyoto Japan; ^2^ Department of Respiratory Medicine Hikone Municipal Hospital Hikone Shiga Japan

**Keywords:** blood culture, co‐infection, community‐acquired pneumonia, *Klebsiella variicola*, *Streptococcus pneumoniae*

## Abstract

Community‐acquired pneumonia (CAP) remains a leading cause of morbidity and mortality. *Streptococcus pneumoniae* is the most common causative pathogen, with urinary antigen testing aiding the diagnosis thereof; however, blood culture yields are low, and guideline recommendations vary. We herein report a case of a 77‐year‐old man with pneumococcal pneumonia confirmed by urinary antigen testing whose blood cultures also revealed *Klebsiella variicola* . The detection of this co‐infection would have been missed without blood cultures. Moreover, this combination of pathogens is first reported. This case underscores the importance of routine blood cultures in CAP diagnosis and supports the Japanese Respiratory Society's recommendation for their use in hospitalised patients.

## Graphical abstract

We report a case of community‐acquired pneumonia. In this case, the blood culture was essential for detection of co‐infection of *Streptococcus pneumoniae* and *Klebsiella variicola* , which combination is first reported.

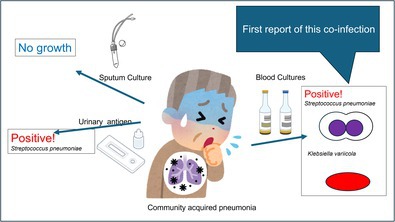

## 1 Introduction

Community‐acquired pneumonia (CAP) is a common infectious disease, with an estimated annual incidence of approximately 1.88 million cases in Japan. The fatality rate of CAP remains approximately 2%–4%, and pneumonia accounts for the fourth leading cause of death in Japan. *Streptococcus pneumoniae* is the most frequently identified causative pathogen of CAP, followed by *Haemophilus influenzae* , *Staphylococcus aureus* , and *Mycoplasma pneumoniae* in Japan. Identifying the causative pathogen is fundamental for choosing effective antibiotic therapy. National guidelines differ regarding the recommended microbiological examinations at the time of admission for patients with CAP [1, 2, 3], and the Japanese Respiratory Society (JRS) recommends routine blood cultures for hospitalised patients [1]. Herein, we report a case of CAP caused by co‐infection with *S. pneumoniae* and *Klebsiella variicola* , which could not have been diagnosed without blood cultures. This case emphasises the importance of performing blood cultures for accurate diagnosis and appropriate management of CAP, supporting the JRS guideline recommendations.

## 2 Case Report

**FIGURE 1:**
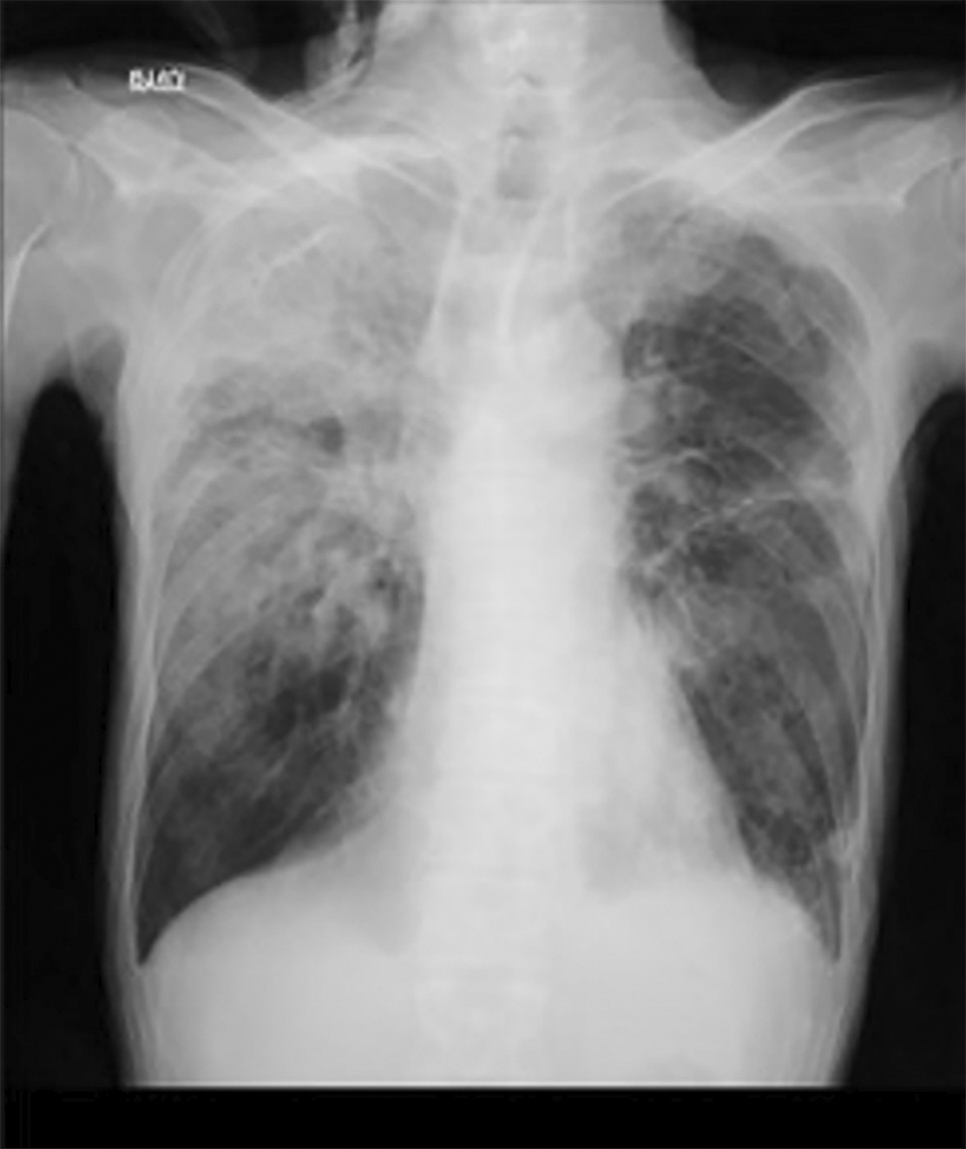
Chest radiography at initial examination. Chest radiography showing an infiltrative shadow in the right lung field, raising suspicion of pneumonia and prompting referral for further evaluation.

**FIGURE 2:**
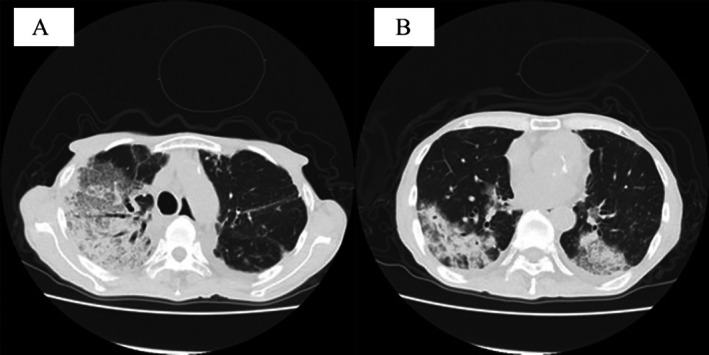
Computed tomography demonstrates consolidation involving the right upper to lower lobes (A), with additional consolidation noted in the left lower lobe (B). (A) CT image demonstrating extensive consolidation extending from the right upper lobe to the right lower lobe. (B) Additional consolidation in the left lower lobe indicating multilobar involvement in both lungs.

A 77‐year‐old man with well‐controlled type 2 diabetes and untreated *mycobacterium avium* complex lung disease presented to a family clinic with progressive dyspnoea. Chest radiography revealed an infiltrative shadow in the right lung field (Figure 1), and the patient was referred to the emergency department for further investigation. Laboratory tests revealed leukopenia and marked elevation of inflammatory markers (Table 1), while chest computed tomography (CT) revealed consolidation involving the right upper to lower lobes, with additional consolidation in the left lower lobe (Figure 2A,B), leading to a diagnosis of lobar pneumonia. Upon admission, the urinary pneumococcal antigen test result was positive, leading to a diagnosis of *S. pneumoniae* pneumonia. Urine testing and CT scans ruled out other sources of infection, such as urinary or biliary tract infections. Sputum cultures did not reveal any significant bacterial findings. Empirical antimicrobial therapy with 2 g of ceftriaxone (CTRX) once daily was initiated for *S. pneumoniae* pneumonia. Blood cultures obtained at admission were collected in two sets (aerobic and anaerobic bottles), both of which tested positive for *S. pneumoniae* and *K. variicola* several days later. The antimicrobial susceptibility results are presented in Table 2. CTRX was continued, and gradual improvements in both respiratory function and overall condition were achieved. The antibiotic regimen was subsequently switched to oral levofloxacin, and antimicrobial therapy was completed after 14 days, in accordance with the JRS guidelines. The patient subsequently recovered and was discharged.

**TABLE 1:** Laboratory findings on admission compared with those 2 months prior to admission.

	Result	
	On admission	Two months prior to admission
White blood cell count (/μL)	3300	4000
Haemoglobin (g/dL)	13.0	11.8
Platelet count (×10^4^/μL)	10.3	25.6
C‐reactive protein (mg/dL)	28.87	0.09
Total protein (mg/dL)	5.7	5.8
Albumin (mg/dL)	3.1	3.4
Total bilirubin (mg/dL)	2.2	0.5
Aspartate aminotransferase (U/L)	50	73
Alanine aminotransferase (U/L)	34	65
Alkaline phosphatase (U/L)	67	104
Blood urea nitrogen (mg/dL)	68	30
Creatinine (mg/dL)	1.35	0.90
Hb A_1c_ (%)	6.4	8.0

Abbreviation: Hb, Haemoglobin.

**TABLE 2:** Antimicrobial susceptibility results of *Streptococcus pneumoniae* and *Klebsiella variicola* isolated from blood cultures.

Antimicrobial agent	*Streptococcus pneumoniae*		*Klebsiella variicola*	
	MIC (μg/mL)	Interpretation	MIC (μg/mL)	Interpretation
Penicillin G			≤ 0.0625	S
Ampicillin	≥ 32	R		
Piperacillin	≥ 128	R		
Amoxicillin/clavulanic acid			≤ 0.25	S
Ampicillin/sulbactam	≥ 32	R		
Cefazolin	≤ 2	S		
Cefuroxime	≤ 2	S	≤ 0.25	S
Cefmetazole	≤ 8	S		
Cefotiam	≤ 1	S	≤ 0.25	S
Ceftriaxone	≤ 0.5	S	≤ 0.25	S
Ceftazidim	≤ 0.5	S		
Cefepime	≤ 1	S	≤ 0.25	S
Imipenem/cilastatin	≤ 0.25	S	≤ 0.0625	S
Meropenem	≤ 0.125	S	≤ 0.0625	S
Amikacin	≤ 4	S		
Gentamicin	≤ 2	S		
Erythromycin			≤ 0.0625	S
Clindamycin			≤ 0.0625	S
Minocycline	≥ 16	R		
Vancomycin			≤ 0.25	S
Levofloxacin	≤ 0.25	S	1	S
Trimethoprim‐sulfamethoxazole	≤ 1	S	1	1

Abbreviations: I, intermediate; R, resistant; MIC, minimum inhibitory concentration; S, susceptible.

## 3 Discussion

We report a case of CAP caused by co‐infection with *S. pneumoniae* and *K. variicola* , which could not have been diagnosed without obtaining blood cultures. Moreover, to the best of our knowledge, concurrent bacteraemia caused by these two organisms has not previously been reported.

*S. pneumoniae* is the most frequently identified causative organism of CAP. Urinary antigen testing for *S. pneumoniae* is a widely used rapid diagnostic tool because of its convenience, speed, and relatively high sensitivity and specificity. However, this test detects only pneumococcal antigens and cannot identify additional pathogens or provide information on antimicrobial susceptibility. In clinical practice, a positive urinary antigen result often leads clinicians to initiate or maintain narrow‐spectrum empirical therapy, assuming monomicrobial pneumococcal pneumonia. No pathogen was detected in the sputum in the present case, and if blood cultures had not been obtained, the patient might have been treated for pneumonia caused only by *S. pneumoniae* . When treating suspected pneumococcal pneumonia, standard practice involves initiating therapy with CTRX in consideration of the possibility of penicillin‐resistant pneumococci, followed by de‐escalation if the clinical course is favourable. However, de‐escalation of antibiotic therapy based solely on urinary antigen results might have led to treatment failure. This is because, in Japan, even penicillin‐resistant *S. pneumoniae* isolates exhibit relatively low minimum inhibitory concentrations of amoxicillin or ampicillin, to which *Klebsiella* species are naturally resistant; these are recommended as first‐line agents for *S. pneumoniae* pneumonia [1]. Fortunately, in the present case, levofloxacin was selected as the first‐line therapy based on blood culture results, allowing us to avoid the aforementioned de‐escalation. Furthermore, while the duration of antimicrobial therapy is trending towards shorter courses worldwide [4, 5], treatment duration may be extended in cases of positive blood cultures, making blood culture a meaningful test for patients with community‐acquired pneumonia.

The extent of microbiological investigations performed for CAP differs considerably among the guidelines. While the JRS guidelines recommend routine sputum examination for hospitalised patients with pneumonia, the American Thoracic Society (ATS) guidelines do not recommend routine sputum testing in cases where empirical therapy does not cover drug‐resistant pathogens [2], and the British Thoracic Society (BTS) guidelines advise against its routine use except in moderate‐to‐severe cases [3]. Similarly, urinary antigen testing, recommended by the JRS [1], is limited to severe cases in the ATS guidelines [2] and moderate‐to‐severe cases in the BTS guidelines [3]. The JRS recommends blood cultures for all hospitalised patients, whereas the ATS restricts them to severe cases requiring coverage for drug‐resistant pathogens, and the BTS limits them to moderate‐to‐severe disease [1, 2, 3]. Although the positivity rate of blood cultures in CAP is as low as 5%–14%, positive findings can significantly contribute to targeted therapy. The present case demonstrates that blood cultures remain indispensable for the accurate diagnosis and appropriate management of CAP, supporting the JRS guidelines. This case reinforces our understanding that blood cultures are essential tests for cases of community‐acquired pneumonia requiring hospitalisation, particularly considering the possibility of unexpected mixed infections.

In CAP, polymicrobial infections have been reported in approximately 14% of cases [6], with the most frequent bacterial combinations being *S. pneumoniae* with *H. influenzae* and *S. pneumoniae* with *S. aureus* [6]. In the present case, *K. variicola* was detected alongside *S. pneumoniae* . *K. variicola* is a novel bacterium belonging to the *K. pneumoniae* complex, originally recognised as a plant‐associated specie. Clinically, the infection has been associated with respiratory tract infections and higher mortality than *K. pneumoniae*, and is often isolated from immunocompromised patients, including those with malignancy, diabetes, or chronic lung disease [7]. In the present case, several clinical factors may have predisposed the patient to *K. variicola* infection, including the patient's advanced age, history of chronic lung disease with mild mycobacterial infection, and diabetes diagnosis. To the best of our knowledge, this is the first report of pneumonia caused by co‐infection with *S. pneumoniae* and *K. variicola* .

In summary, we encountered a rare case of pneumonia caused by co‐infection with *S. pneumoniae* and *K. variicola* . This case highlights the importance of blood cultures in the management of CAP and supports the JRS guidelines.

## Author Contributions

N.F. and I.O.: conceptualization, writing the original draft, and visualisation. K.F., S.Y., T.I.1, T.I.2, O.K., and K.T.: investigation. K.T.: supervision. All authors have reviewed and revised the manuscript for intellectual content. All the authors approved the final version of the manuscript.

## Funding

The authors have nothing to report.

## Consent

The authors declare that written informed consent was obtained for the publication of this manuscript and accompanying images and attest that the form used to obtain consent from the patient complies with the Journal requirements as outlined in the author guidelines.

## Conflicts of Interest

Kohei Fujita is an Editorial Board member of Respirology Case Reports and a co‐author of this article. He was excluded from all editorial decision‐making related to the acceptance of this article for publication. The other authors declare no conflicts of interest.

## Data Availability

The data that support the findings of this study are available from the corresponding author upon reasonable request.
